# The Influence of Two-Component Mixtures from Spanish Origanum Oil with Spanish Marjoram Oil or Coriander Oil on Antilisterial Activity and Sensory Quality of a Fresh Cut Vegetable Mixture

**DOI:** 10.3390/foods9121740

**Published:** 2020-11-26

**Authors:** Karolina Kraśniewska, Olga Kosakowska, Katarzyna Pobiega, Małgorzata Gniewosz

**Affiliations:** 1 Department of Food Biotechnology and Microbiology, Institute of Food Sciences, Warsaw University of Life Sciences−SGGW (WULS-SGGW), Nowoursynowska 159, 02-776 Warsaw, Poland; katarzyna_pobiega@sggw.edu.pl (K.P.); malgorzata_gniewosz@sggw.edu.pl (M.G.); 2 Department of Vegetable and Medicinal Plants, Institute of Horticultural Sciences, Warsaw University of Life Sciences–SGGW (WULS-SGGW), Nowoursynowska 159, 02-776 Warsaw, Poland; olga_kosakowska@sggw.edu.pl

**Keywords:** essential oils, antibacterial activity, *Listeria monocytogenes*, minimally processed vegetables, food safety

## Abstract

The aim of this study was to evaluate two-component mixtures of essential oils (EOs) in sub-inhibitory concentrations for antilisterial protection of minimally processed vegetables. Spanish origanum oil (SOO), Spanish marjoram oil (SMO) and coriander oil (CO) and a mixture of fresh leafy vegetables with red beet were used. The chemical compositions of selected EOs were analyzed by gas chromatography. Carvacrol was the major component of SOO. The dominant active component of SMO was 1,8-cineole, while the major constituent in CO was linalool. The study shows that essential oils in combination, namely SOO + SMO and SOO + CO in a ¼ MIC + ¼ MIC (Minimal Inhibitory Concentration), have a synergistic effect against *L. monocytogenes*. The reduction of *L. monocytogenes* in vegetable mixtures treated by EOs in these selected combinations after 24 h of storage was approximately equal to the limit of detection. Furthermore, it was found that the vegetable mixture treated with SOO + SMO had the highest-rated sensorial quality and could therefore meet consumer expectations.

## 1. Introduction

Listeriosis caused by *Listeria monocytogenes* is still a rare disease in humans; however, due to high pathogenicity, the need for hospitalization and high mortality, especially among the elderly, it is one of the most dangerous foodborne diseases [1,2]. In the years 2009–2018, an increase in listeriosis in the EU/EEA was observed [3]. High-risk foods due to the presence of *L. monocytogenes* include ready-to-eat (RTE) products, particularly RTE smoked and gravad fish, heat-treated meat and soft and semi-soft cheese. Thus far, fruits and vegetables have been classified as “low risk” products [3,4,5,6,7]. However, currently, foodborne outbreak statistics show that there is an increasing trend in fresh-cut produce and associated outbreaks mostly caused by such bacterial agents as *Listeria monocytogenes*, *Salmonella* spp. and *Escherichia coli* O157:H7 [8,9]. Fresh vegetables are foods with a short shelf life, so there is a need to optimize moderate processing and storage [10,11]. Among natural preservatives are plant essential oils, which can be used to disinfect minimally processed vegetables [12]. Essential oils from spice plants have long been used in the food industry as flavorings in food and drink. Essential oils also have a number of biological activities, including antimicrobial, antioxidant, cytotoxic, anti-inflammatory and insecticidal [13]. Even a small addition of essential oils to minimally processed vegetables can enrich them culinarily. In addition, essential oils with strong antimicrobial activity can improve the microbiological safety of these products [14]. It was found that 0.3% and 0.5% cinnamon oil was the most effective against *Salmonella enterica* serotype Newport on organic leafy greens [15], and marjoram essential oil at 0.5% concentration showed disinfection capacity of multidrug-resistant *Salmonella enterica* serovar Schwarzengrund in lettuce [14]. Additionally, oregano oil effectively reduced levels of total mesophilic microorganisms in fresh leaf lettuce and radish sprouts [16]. *Tetraclinis articulata* essential oils can be used as a potential source of fungicides to protect stored tomatoes from pathogens and saprophytic fungi [17]. However, the disadvantage of essential oils in food production, limiting their widespread use, is the intense odor and taste, not always favorably affecting the sensory characteristics of products. Therefore, in the production of food, essential oils are often used in high dilutions, which in turn may prove ineffective in microbiological food protection. In order to preserve the benefits of using essential oils (culinary and antimicrobial) in the production of low-processed vegetables, combinations of essential oils in reduced concentrations are used, while it is necessary to select such a combination for a specific food product [18]. In the study presented by Lv et al. [19], it was shown that combined selected Eos, e.g., oregano–basil, basil–bergamot, oregano–bergamot and oregano–perilla, were considered antimicrobial agents with higher efficacy compared to EOs used alone. Furthermore, this study also suggested that combinations of EOs could minimize application concentrations and consequently reduce any adverse sensory impact in food.

The purpose of this study was to evaluate the antilisterial efficacy of two-component mixtures of essential oils in sub-inhibitory concentrations in model research and on a mixture of fresh low-processed vegetables and to assess the sensory properties of a mixture of vegetables treated with essential oils and their mixtures.

## 2. Materials and Methods

### 2.1. Materials

Essential oils (EOs): Spanish origanum oil of *Thymbra capitata* (L.) Cav., coriander oil of *Coriandrum sativum* L., Spanish marjoram oil of *Thymus mastichina* (L.) L. from Sigma Aldrich (Darmstadt, Germany). Chemical reagents: dimethylsulfoxide (DMSO) and sodium hypochlorite bought from Chempur (Piekary Śląskie, Poland), phosphate-buffered saline (PBS), tryptic soybean broth (TSB), tryptic soy agar (TSA), buffered peptone water (BPW) and glycerol from BTL (Łódź, Poland), Palcam Agar plus selective supplement (PA) bought from Merck (Darmstadt, Germany).

Plant materials: fresh endive chicory (*Cichorium endivia* L.), lettuce (*Lactuca sativa* L.) and red beet (*Beta vulgaris* L.) and a commercial mixture of the same cut and ready-to-eat vegetables were purchased at the local store on delivery and stored at 8 ± 1 °C.

The bacterial strain of *Listeria monocytogenes* 17/11 (a clinical isolate taken from a patient with listeriosis) was from the National Institute of Public Health in Warsaw (Warsaw, Poland). The strain was stored at −80 °C in 25% glycerol. The frozen strain was transferred to TSA and incubated at 37 °C for 24 h. Then, the culture was transferred to PBS to reach a population of approximately 1 × 10^8^ CFU mL^−1^. The solution was tenfold diluted to 1 × 10^7^ CFU mL^−1^.

### 2.2. GC-FID Analysis

The quantitative GC-FID (gas chromatograph coupled with a flame ionization detector) analysis was carried out using a Hewlett Packard 6890 gas chromatograph equipped with a flame ionization detector (FID) and capillary, polar column HP 20M (25 m × 0.32 mm × 0.3 µm film thickness). The analysis was performed using the following temperature program: oven temperature isotherm at 60 °C for 2 min, then it was programmed from 60 to 220 °C at a rate of 4 °C per min and held isothermal at 220 °C for 5 min. Injector and detector temperatures were, respectively, at 220 and 260 °C. The carrier gas (He) flow was 1.1 mL min^−1^. The split ratio was 1:500. Manual injection of 0.3 µL of essential oil was applied. Component identification was confirmed by comparison of their retention times with those of pure authentic samples and by means of their linear retention indices (RI) relative to the series of *n*-hydrocarbons (C7-C30), under the same operating conditions. RI were compared with those reported in the literature [20]. The percentage composition of the essential oils was computed by the normalization method from the GC peak areas, without the use of correction factors.

### 2.3. Determination of the Minimal Inhibitory Concentration (MIC)

The EOs were dissolved in 10% DMSO, and then their solutions from 0.05% to 1.5% in TSB were prepared. Mixtures of two EOs in TSB were also prepared according to Table 1.

Next, 380 μL of TSB containing EOs separately or mixtures at different concentrations and 20 μL of inoculum *L. monocytogenes* were transferred to wells of a sterile Honeycomb plate (Thermo Scientific, Warsaw, Poland). In each well, the initial number of *L. monocytogenes* was approximately 5 × 10^5^ CFU mL^−1^. Strain growth control was performed in TBS (without EO) with the *L. monocytogenes* inoculum. The negative control was TBS without EO and bacterial inoculum. The Honeycomb 2 plates were placed on a Bioscreen C apparatus (OY Growth Curves Ab Ltd., Turku, Finland) and incubated at 37 °C for 24 h. Plates were shaken before measurement and the automatic optical density (OD) was measured at λ 600 nm every 1 h. A smaller change in OD value than 0.2 (∆OD < 0.2) at the initial and final incubation hours was considered as no test strain growth. The minimum inhibitory concentration (MIC) of the EO or mixture of EOs was defined as the lowest concentration at which there was no growth of the test strain. Three independent series of experiments in triplicate were performed.

Antilisterial activity of EO mixtures was expressed as the fractional inhibitory concentration index (FICI), equal to the sum of fractional inhibitory concentrations (FICs) for each EO, which was calculated according to the following formula [21]:

FICI = FIC_EO1_ + FIC_EO2_ = (MIC_EO1_ in combination/MIC_EO1_ alone) + (MIC_EO2_ in combination/MIC_EO2_ alone)

The FIC is calculated by comparing the MIC of each EO alone with the combination-derived MIC. The synergistic effect of EOs in the mixture is at FICI values ≤ 0.5, nonsynergistic or additive action is at values in the range 1 ≥ FICI > 0.5, and FICI values between 1 and 4 indicate a neutral effect. A FICI > 4 indicates antagonistic activity of EOs in the mixture [22].

### 2.4. Microbiological Analyses

#### 2.4.1. In Vegetable Filtrate

In total, 200 g of vegetable mixture in equal proportions of endive chicory, lettuce and red beet was washed under running water and blended with 800 mL of distilled water and filtered through gauze. Then, the vegetable filtrate was sterilized at 117 °C for 15 min. Its pH was 6.27. Five 20 mL portions of vegetable filtrate were prepared with EOs in MIC and with two mixtures of SOO + CO and SOO + SMO at ¼ MIC + ¼ MIC combination. The control sample was 20 mL of vegetable filtrate without EOs. To the samples with/without EOs, 1 mL of *L. monocytogenes* inoculum (prepared as above) was added and the samples were incubated at 37 °C for 24 h. The cell numbers of *L. monocytogenes* were determined at 0, 4, 6, 12 and 24 h in TSA. Plates were incubated at 37 °C for 24 h. The result was reported in CFU × mL^−1^. Three independent series of experiments in triplicate were performed.

#### 2.4.2. In a Mixture of Fresh Cut Vegetables

A 600 g commercial ready-to-eat mixture of fresh cut vegetables (endive chicory, lettuce and red beet in equal proportions) was first washed under running water, then washed in distilled water and immersed in a solution of 0.05% sodium hypochlorite for 15 min to reduce natural microflora. Then, the vegetables were immersed in the inoculum of *L. monocytogenes* (prepared as above) at room temperature (22 °C) for 15 min. They were air-dried at 22 °C for 1 h in the biosafety cabinet for better cell adhesion, with plant tissue and excess fluid removed. The vegetables were aseptically divided into six portions of 50 g, in duplicate, which for 1 min were immersed in solutions containing SOO, CO or SMO at MIC (0.1%, 0.4% and 0.9%, respectively) prepared in phosphate-buffered saline (PBS, pH 7.0) or mixtures of SOO + CO and SOO + SMO in a combination at ¼ MIC + ¼ MIC, previously diluted in 10% DMSO. The control sample consisted of inoculated vegetables dipped in sterile distilled water. Thirty minutes after sample preparation, the number of *L. monocytogenes* was determined. For this purpose, 25 g samples were transferred to 225 mL of BPW and homogenized in a stomacher (Lab-Blender 400, Seward Medical, London, UK) for 1 min. All samples were diluted ten-fold and transferred in duplicate into PA. Plates were incubated at 37 °C for 24 h. Three independent series of experiments in triplicate were performed.

### 2.5. Sensory Evaluation

A commercial ready-to-eat mixture of vegetables (described above) was used. The vegetables were soaked in solutions of EOs prepared in phosphate-buffered saline (PBS, pH 7.0). The solutions contained EOs alone at MIC, i.e., 0.1% SOO, 0.4% CO, 0.9% SMO, or mixtures of SOO + CO and SOO + SMO at ¼ MIC + ¼ MIC, previously diluted in 10% DMSO. The vegetables were incubated in solutions for 1 min, after which they were dried and stored in sterile plastic containers at 8 ± 1 °C and 55%–60% relative humidity in a cooling incubator (CHL5, Pol-Eko-Aparatura, Wodzisław, Poland) for 4 days. All samples were evaluated after 30 min and after 4 days of refrigerated storage. The sensory evaluation of vegetables was performed by a team of 50 partially trained panelists, consisting of employees and students of the Institute of Food Sciences, WULS-SGGW in Warsaw, using a nine-point hedonic scale in accordance with the methodology described by Baryłko-Pikielna and Matuszewska [23]. Samples of 10 g were coded with random numbers and given in randomized duplicates. The panelists used a list of qualitative descriptors established at a special session, i.e., color, texture, odor, taste and overall quality, which is a summary of the features included in the assessment. The intensity of each descriptor was assessed on a continuous graphic scale, with edge markings, where 1 represents extremely dislike and 9 represents extremely like. Average values were calculated and the results were presented in the form of a chart.

### 2.6. Statistical Analysis

All the measurements were performed at least in duplicate. One-way ANOVA was used for independent samples. In order to verify the significance of differences between the means, Tukey’s test was used at the significance level α = 0.05. Statistical analysis was performed using Statistica version 10.

## 3. Results

### 3.1. Chemical Composition of Essential Oils

Table 2 shows the chemical composition of SOO, CO and SMO. Twelve compounds were found in SOO, with the highest percentage of carvacrol (79.11%), linalool (4.14%) and *p*-cymene (4.01%). CO contained 15 compounds and was rich in linalool (68.45%), α-pinene (6.66%) and *p*-cymene (6.14%). In turn, SMO contained 14 compounds, among which 1,8-cineole (69.15%) had the largest share, followed by β-pinene (4.73%) and limonene (4.09%). EOs contained only three of the same components: α-pinene, *p*-cymene and linalool.

### 3.2. Inhibition of L. monocytogenes Growth by Essential Oils

**Figure 1:**
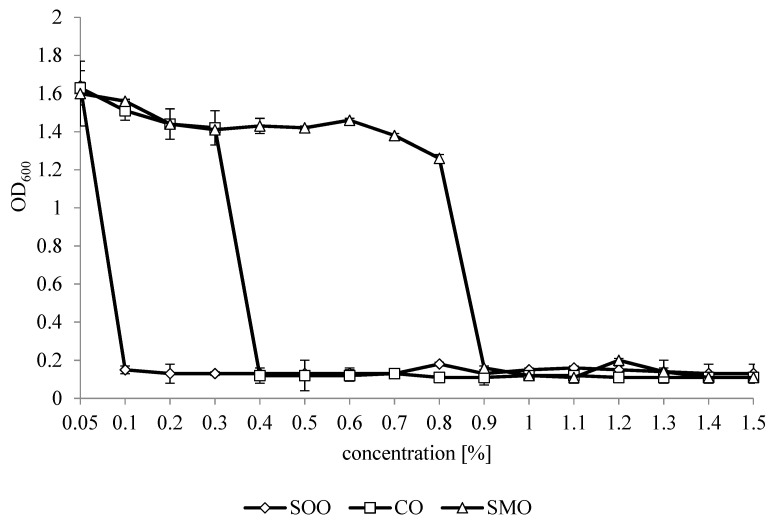
Changes in OD_600_ for an *L. monocytogenes* culture with essential oils after 24 h. SOO—Spanish origanum oil, CO—coriander oil, SMO—Spanish marjoram oil.

Figure 1 shows the effect of EOs in concentrations in the range from 0.05% to 1.5% on changes in optical density (OD) of *L. monocytogenes* after 24 h. The essential oils’ MIC values for *L. monocytogenes* varied from 0.1% to 0.9%. The smallest MIC value was found for SOO (0.1%), followed by CO (0.4%) and the highest for SMO (MIC 0.9%).

### 3.3. Inhibition of L. monocytogenes Growth by Mixtures of Essential Oils

**Figure 2:**
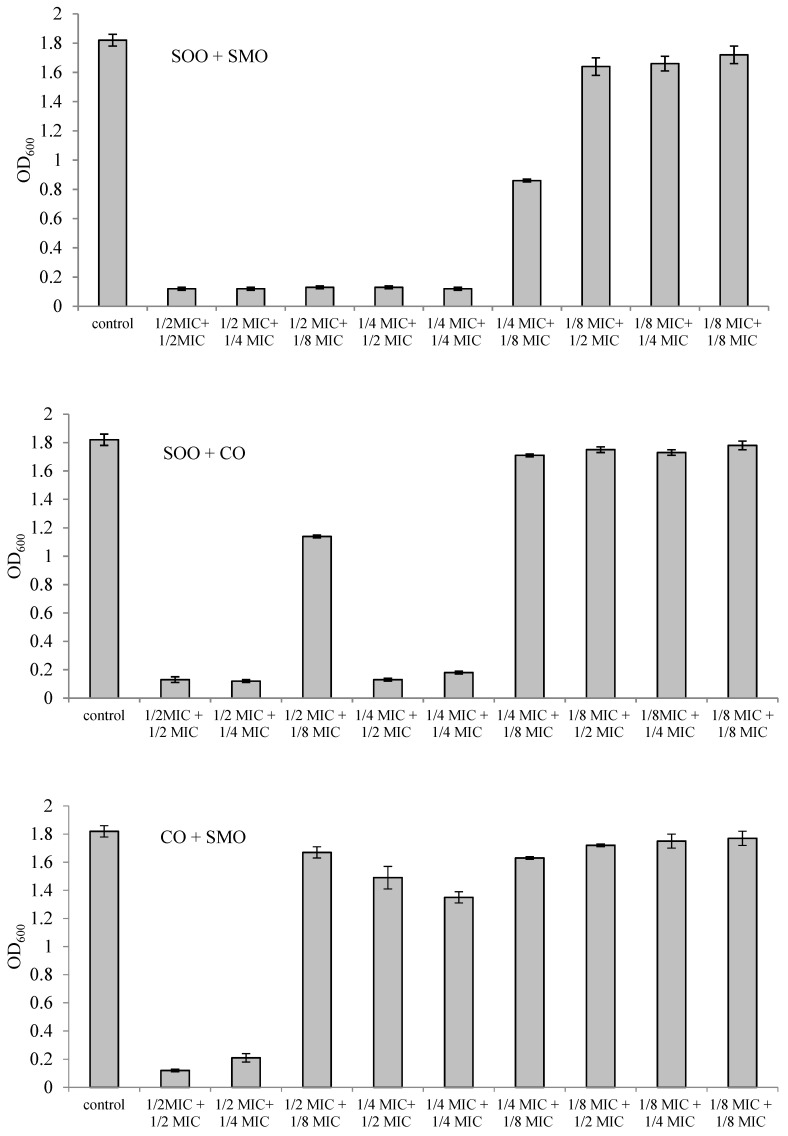
Changes in OD_600_ for an *L. monocytogenes* culture with mixtures of essential oils (EOs) after 24 h. SOO—Spanish origanum oil, SMO—Spanish marjoram oil, CO—coriander oil.

The effect of mixtures of two EOs in different combinations and concentrations on inhibition of *L. monocytogenes* growth is shown in Figure 2. Five SOO + SMO mixtures showed bacteriostatic activity against *L. monocytogenes*. These were mixtures of SOO at ½ MIC in combination with SMO at ½ MIC, ¼ MIC or ⅛ MIC and mixtures of SOO at ¼ MIC in combination with SMO at ½ MIC or ¼ MIC. Four mixtures of SOO + CO inhibited the growth of *L. monocytogenes*, in which SOO at ½ MIC was in combination with CO at ½ MIC or ¼ MIC and SOO at ¼ MIC was in combination with CO at ½ MIC or ¼ MIC. On the other hand, only two mixtures of CO + SMO showed a bacteriostatic effect against *L. monocytogenes*. These were mixtures containing CO + SMO in a ½ MIC + ½ MIC combination and in a ½ MIC + ¼ MIC combination.

A FICI value of 0.5 (Table 3) was observed only for two mixtures, SOO + SMO and SOO + CO in a ¼ MIC + ¼ MIC combination, suggesting a synergistic effect of the components of the EOs in these combinations against *L. monocytogenes*. The FICI value of 0.75 revealed the additive effect of SOO + SMO and SOO + CO in a ½ MIC + ¼ MIC combination. The SOO + SMO mixture in the combination ½ MIC + ⅛ MIC also had an additive effect (FICI 0.625). In turn, the interaction of essential oils in SOO + SMO, SOO + CO and CO + SMO mixtures in a ½ MIC + ½ MIC combination was neutral in relation to *L. monocytogenes*. The combination of CO and SMO at sub-inhibitory concentrations (¼ MIC + ¼ MIC or ¼ MIC + ⅛ MIC) did not inhibit the growth of *L. monocytogenes*.

### 3.4. Inactivation of L. monocytogenes in Vegetable Filtrate and Mixture of Fresh Cut Vegetables with EOs

The inactivation of *L. monocytogenes* in the vegetable filtrate with EOs at 1 MIC alone or with the mixtures of EOs at ¼ MIC + ¼ MIC is shown in Table 4. The initial number of *L. monocytogenes* in the control was 5.8 log CFU mL^−1^ and increased by three log cycles after 24 h. The addition to the vegetable filtrate of SOO at 1 MIC reduced the number of *L. monocytogenes* approximately by five log cycles after 4 h. CO and SMO at 1 MIC had a weaker antilisterial effect, because it reduced the number of bacteria by 3.5 and 2.3 log cycles after 4 h. In the vegetable filtrate with the addition of EOs alone at 1 MIC, it was found that the number of viable *L. monocytogenes* cells was at the limit of detection (<1 log CFU × mL^−1^) after 24 h.

Adding to the vegetable filtrate EO mixtures at sub-inhibitory concentrations significantly (*p* < 0.05) reduced the number of *L. monocytogenes* after 4 h. However, differences in the potency of the EO mixtures against bacteria were observed. At the same time, the SOO + CO mixture resulted in a greater reduction (*p* < 0.05) in the number of cells in the test strain than the SOO + SMO mixture at a ¼ MIC + ¼ MIC combination. In the sample with the SOO + CO mixture, the number of *L. monocytogenes* decreased by 3.8 log cycles after 4 h and this tendency persisted in the remaining time interval. No significant differences (*p* > 0.05) were found between *L. monocytogenes* numbers in the vegetable filtrate treated with the SOO + CO mixture and CO alone for up to 6 h. The SOO + SMO mixture had a weaker effect on *L. monocytogenes*, which reduced the number of cells approximately by three log cycles after 4 h. After 24 h, the number of *L. monocytogenes* in the vegetable filtrate containing EO mixtures was <1 log CFU × mL^−1^.

Treatment of the fresh cut vegetable mixture by EOs or their mixtures resulted in a decrease in the number of *L. monocytogenes* approximately by three log cycles after 30 min (Table 4). SOO at 0.1% (1 MIC) had very strong activity and reduced the number of *L. monocytogenes* to the limit of detection. Statistically significant differences (*p* < 0.05) were found between bacterial numbers in the CO-treated vegetable mixture at 1 MIC and SOO + CO, at a ¼ MIC + ¼ MIC combination, which indicates the stronger antilisterial effect of this mixture.

### 3.5. Sensory Evaluation of a Mixture of Fresh Cut Vegetables Treated with EOs or EO Mixtures

**Figure 3:**
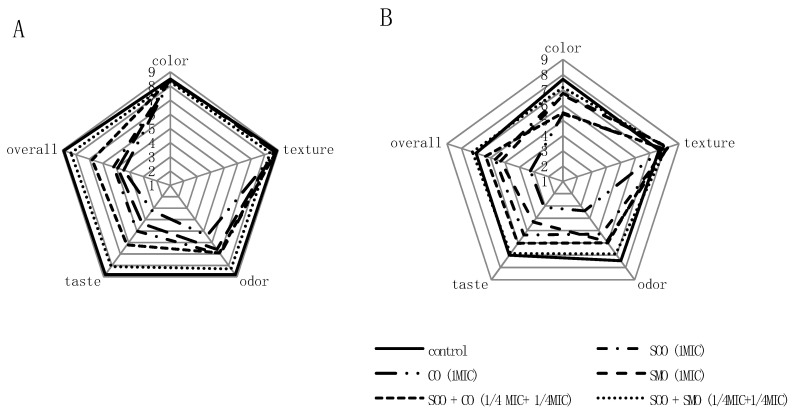
Changes in the sensory quality of a vegetable mixture treated by the addition of EOs or a mixture of EOs (**A**) after 30 min, (**B**) after 4 days of storage.

The results of sensory analysis of the mixture of vegetables treated with EOs at 1 MIC and their mixtures at a ¼ MIC + ¼ MIC combination are shown in Figure 3. Treatment of the vegetable mixture with EOs or SOO + CO and SOO + SMO mixtures did not have a significant effect (*p* > 0.05) on color or texture, but it statistically (*p* < 0.05) significantly affected the odor, taste and overall quality assessment, which reduced the desirability of these vegetables (Figure 3A). Lower scores for these descriptors were obtained for samples treated by EOs alone than by mixtures of EOs. The sample treated with SOO + SMO in a ¼ MIC+ ¼ MIC combination had the highest overall quality. The average scores for all descriptors of this sample did not differ significantly statistically (*p* < 0.05) from the control sample. The CO-treated sample at 1 MIC had the lowest average odor and taste scores, which significantly reduced the desirability of this sample.

After 4 days of storage, the vegetable mixture quality deteriorated (Figure 3B). Vegetable samples treated with EO mixtures were rated better than EOs alone. The control sample and the sample treated with the SOO + SMO mixture at a ¼ MIC + ¼ MIC combination differed the least. Both samples obtained the highest scores, with overall quality of 7.00 and 7.25, respectively.

## 4. Discussion

This research attempted antilisterial protection of vegetables minimally processed by two-component mixtures of essential oils in sub-inhibitory concentrations with simultaneous good sensory evaluation of the product. The results indicate that SOO, SMO and CO had inhibitory effects on *L. monocytogenes* growth, but at different MIC values (SOO < CO < SMO). The antibacterial activity of EOs depends on the phenolic components in which EOs are rich [24]. The SOO had the lowest MIC values, due to the highest percentage of carvacrol (79.11%) [25]. Carvacrol is a phenolic compound that is credited with strong antibacterial properties. Cell death under the influence of carvacrol is the result of a rupture of the cytoplasmic membrane, which consequently leads to leakage of intracellular content [26]. CO and SMO had higher MIC values. Spanish marjoram oil is rich in 1,8-cineole (69.15%), while in coriander oil, there is the most linalool (68.45%). Previous studies have shown lower antilisterial activity of 1,8-cineole, linalool and α-pinene than carvacrol and thymol [27,28,29]. The mechanism of action of monoterpenes found in EOs has also been described; among other things, it can disrupt the cellular respiration in bacteria [30].

Earlier studies suggest that a single treatment is usually not sufficient to ensure microbiological safety of fresh products [29,31]. Based on the FICI value, it was found that the mixtures of SOO + SMO and SOO + CO in a ¼ MIC + ¼ MIC combination had synergistic antilisterial effects, which has not been reported before. In turn, the mixture of SMO + CO in a ¼ MIC + ¼ MIC combination did not inhibit the growth of *L. monocytogenes*. The additive effect of this mixture was observed in a ¼ MIC + ½ MIC combination and was probably the result of summing the essential oils constituents with similar chemical structures. Mixtures of EOs in the other combinations had an additive or indifferent antilisterial effect. Similarly, a mixture of essential oils from *Origanum vulgare* L. and *Rosmarinus officinalis* L. and mixtures of *O. vulgare* with *Thymus vulgaris* (thyme) essential oils had a synergistic or additive effect on *L. monocytogenes* and other foodborne pathogens [32,33]. In earlier studies, a combined antilisterial effect between nisin and EOs was observed, and the addition of a third factor in the form of diglycerol monolaurate led to further combined antilisterial activities between the essential oil constituents and nisin even at lower, sub-lethal concentrations [29].

The *L. monocytogenes* suspension was then exposed to EOs in 1 MIC used alone and in combinations at ¼ MIC doses in the vegetable filtrate [32]. It turned out that in vegetable filtrate, EO mixtures at sub-inhibitory concentrations killed *L. monocytogenes* cells with the same effect as EOs alone at 1 MIC. At the same time intervals, a significantly greater (*p* < 0.05) reduction in *L. monocytogenes* was caused by the mixture of SOO + CO than by SOO + SMO. This phenomenon can be explained by a greater synergy of ingredients in SOO + CO compared to SOO + SMO. It is difficult to explain the synergy of the main components of EOs, because the antimicrobial activity of EOs consists of all components, even those found in very small amounts. In addition, the effectiveness of antibacterial activity can be enhanced by EO components with lipophilic properties, functional group strength and water solubility [32].

Nevertheless, it should be also noted that application EOs in fresh cut vegetables might be less effective than in vegetable filtrate. This can be explained by the worse contact of EOs with plant tissue due to unevenness and roughness of the surface, hydrophobicity and/or hydrophilicity and the presence of secondary antimicrobial metabolites in the composition of the surface [18,32].

The use of EOs to protect against the development of pathogens is only possible if such a product is accepted by consumers. The odor and taste of essential oils have been mostly determined by their chemical composition [24]. SOO with a high content of phenol monoterpenes, mainly carvacrol, had a sharp spicy flavor and taste, while SMO was distinguished by a pleasant, spicy odor and taste. CO displayed the worst characteristics, because it had a sweet, slightly spicy odor and a bitter taste. Mixtures of EOs in a ¼ MIC + ¼ MIC combination added to vegetables were in very low concentrations (SOO 0.0225%, SMO 0.225% and CO 0.1%). The better antilisterial, i.e., the SOO + CO mixture, has poorer sensory characteristics than the SOO + SMO mixture added to fresh vegetables. The odor and taste of SOO + SMO were minimally perceptible compared to the control, making vegetables with this mixture just as acceptable as vegetables without EOs. Similar results were obtained by Yamazaki et al. [29], where nisin and diglycerol monolaurate were used to enhance the antilisterial activity of essential oils. Such combinations allow for a reduction of the dosage of EO used in food preservation and thereby reduce the development of undesirable flavors in the final product.

## 5. Conclusions

Studies have shown that mixtures of EOs in sub-inhibitory concentrations inactivate *L. monocytogenes* in vegetable filtrate and in minimally processed vegetables. Optimal combinations of essential oils based on SOO + SMO in low concentrations assist in achieving antilisterial activity in final products. Furthermore, such combinations of EOs do not affect the deterioration of odor and taste and maintain acceptable levels of sensory value.

## Figures and Tables

**Table 1:** Two-component mixtures of essential oils in TSB.

Sub-Inhibited Concentration	Mixture of EOs		
	SOO + SMO	SOO + CO	CO + SMO
	(%)		
½ MIC + ½ MIC	0.05 + 0.45	0.05 + 0.2	0.2 + 0.45
½ MIC + ¼ MIC	0.05 + 0.225	0.05 + 0.1	0.2 + 0.225
½ MIC + ⅛ MIC	0.05 + 0.1125	0.05 + 0.05	0.2 + 0.1125
¼ MIC + ½ MIC	0.025 + 0.45	0.025 + 0.2	0.1 + 0.45
¼ MIC + ¼ MIC	0.025 + 0.225	0.025 + 0.1	0.1 + 0.225
¼ MIC + ⅛ MIC	0.025 + 0.1125	0.025 + 0.05	0.1 + 0.1125
⅛ MIC + ½ MIC	0.0125 + 0.45	0.0125 + 0.2	0.05 + 0.45
⅛ MIC + ¼ MIC	0.0125 + 0.225	0.0125 + 0.1	0.05 + 0.225
⅛ MIC + ⅛ MIC	0.0125 + 0.1125	0.0125 + 0.05	0.05 + 0.1125

TSB—tryptic soy agar, EOs—essential oils, MIC—minimal inhibitory concentration, SOO—Spanish origanum oil, SMO—Spanish marjoram oil, CO—coriander oil.

**Table 2:** Gas chromatographic composition (% peak area) of Spanish origanum oil (SOO), coriander oil (CO) and Spanish marjoram oil (SMO) (% of total).

No	SOO			CO			SMO		
	Compound	RI	%	Compound	RI	%	Compound	RI	%
1	α-Pinene	1028	0.54 ± 0.15	α-Pinene	1028	6.66 ± 0.16	α-Pinene	1028	3.08 ± 0.04
2	β-Myrcene	1168	1.11± 0.00	Camphene	1075	1.17 ± 0.02	Camphene	1075	1.35 ± 0.01
3	α-Terpinene	1182	0.97 ± 0.01	β-Pinene	1113	0.45 ± 0.00	β-Pinene	1113	4.73 ± 0.02
4	γ-Terpinene	1249	3.44 ± 0.01	Limonene	1203	2.62 ± 0.04	Sabinene	1124	3.95 ± 0.01
5	*p*-Cymene	1272	4.01 ± 0.01	γ-Terpinene	1248	0.84 ± 0.01	Limonene	1203	4.09 ± 0.01
6	Linalool	1541	4.14 ± 0.00	*p*-Cymene	1272	6.14 ± 0.09	1,8-Cineole	1209	69.15 ± 0.17
7	Bornyl acetate	1580	0.87 ± 0.03	Geraniol	1436	1.13 ± 0.01	*p*-Cymene	1272	3.34 ± 0.02
8	β-caryophyllene	1593	1.10 ± 0.01	Citronellal	1482	0.96 ± 0.03	Camphor	1509	1.21 ± 0.01
9	α-Terpineol	1696	0.77 ± 0.06	unidentified		0.81 ± 0.01	Linalool	1541	1.70 ± 0.01
10	Geranial	1730	2.79 ± 0.01	unidentified		4.79 ± 0.02	Terpinen-4-ol	1584	0.41 ± 0.008
11	Thymol	2165	0.69 ± 0.10	Linalool	1541	68.45 ± 0.27	β-Caryophyllene	1593	0.63 ± 0.008
12	Carvacrol	2213	79.11 ± 0.64	unidentified		0.41 ± 0.01	α-Terpineol	1696	3.81 ± 0.08
13				Geranyl acetate	1751	2.77 ± 0.06	Borneol	1699	1.39 ± 0.01
14				Cirtronellol	1767	0.48 ± 0.02	Caryophyllene oxide	1955	0.67 ± 0.02
15				unidentified		1.13 ± 0.04			
	Total identified		99.54 ± 0.56	Total identified		98.82 ± 0.58	Total identified		99.52 ± 0.28

RI: retention indices relative, values are means ± standard deviations (*n* = 3); SOO—Spanish oreganum oil, SMO—Spanish marjoram oil, CO—coriander oil.

**Table 3:** Fractional inhibitory concentration index (FICI) values and interactions between combinations of essential oils.

Mixture of Essential Oil	½ MIC + ½ MIC	½ MIC + ¼ MIC	½ MIC + ⅛ MIC	¼ MIC + ½ MIC	¼ MIC + ¼ MIC
SOO + SMO	1(I)	0.75(A)	0.625(A)	0.75(A)	0.5(S)
SOO + CO	1(I)	0.75(A)	-	0.75(A)	0.5(S)
CO + SMO	1(I)	-	-	0.75(A)	-

MIC—minimal inhibitory concentration; A—addition; I—indifference; S—synergism; -—not detected. SOO—Spanish origanum oil, SMO—Spanish marjoram oil, CO—coriander oil.

**Table 4:** Changes in the number of *L. monocytogenes* in vegetable filtrate and in mixture of fresh cut vegetables.

Time [h]	Control	SOO 1MIC: 0.1%	CO 1MIC: 0.4%	SMO 1MIC: 0.9%	¼ MIC SOO + ¼ MIC CO	¼ MIC SOO + ¼ MIC SMO
Vegetable filtrate (log_10_ CFU × mL^−1^)						
0	5.8 ± 0.4 ^a^	5.9 ± 0.3 ^a^	5.9 ± 0.1 ^a^	6.1 ± 0.1 ^a^	5.9 ± 0.1 ^a^	6.1 ± 0.2 ^a^
4	6.0 ± 0.1 ^e^	<1.00 ^a^	2.4 ± 0.3 ^c^	3.8 ± 0.2 ^d^	1.8 ± 0.1 ^b^	2.7 ± 0.3 ^c^
6	6.5 ± 0.3 ^e^	<1.00 ^a^	2.2 ± 0.2 ^c^	3.6 ± 0.1 ^d^	1.5 ± 0.1 ^b^	2.1 ± 0.1 ^c^
12	6.7 ± 0.1 ^c^	<1.00 ^a^	2.0 ± 0.1 ^b^	2.3 ± 0.1 ^b^	< 1.00 ^a^	1.9 ±0.1 ^b^
24	8.7 ± 0.2 ^b^	<1.00 ^a^	<1.00 ^a^	<1.00 ^a^	<1.00 ^a^	<1.00 ^a^
Fresh vegetable (log_10_ CFU × g)						
0.5	6.3 ± 0.2 ^e^	<1.00 ^a^	3.8 ± 0.2 ^c^	4.5 ± 0.2 ^d^	3.0 ± 0.2 ^b^	3.7 ± 0.2 ^c^

SOO—Spanish origanum oil, CO—coriander oil, SMO—Spanish marjoram oil. Values are mean ± SD of three separate experiments. Different superscript letters within the same row indicate significant (*p* < 0.05) differences according to Tukey test.
